# Real-world study of antiresorptive-related osteonecrosis of jaw based on the US food and drug administration adverse event reporting system database

**DOI:** 10.3389/fphar.2022.1017391

**Published:** 2022-10-19

**Authors:** Jing Peng, Hui Wang, Zhen Liu, Zhen-Liang Xu, Mei-Xia Wang, Qi-Miao Chen, Ming-Li Wu, Xiao-Lei Ren, Qiu-Hua Liang, Fu-Peng Liu, Bo Ban

**Affiliations:** ^1^ Department of Pharmacy, Affiliated Hospital of Jining Medical University, Jining, Shandong, China; ^2^ Pharmacy Intravenous Admixture Services, Affiliated Hospital of Jining Medical University, Jining, Shandong, China; ^3^ High-School Student, Grade 10, Jining Haida Xingzhi School, Jining, Shandong, China; ^4^ Data Center, Affiliated Hospital of Jining Medical University, Jining, Shandong, China; ^5^ Department of Endocrinology, Affiliated Hospital of Jining Medical University, Jining, Shandong, China

**Keywords:** antiresorptive drugs, osteonecrosis of jaw, romosozumab, the US food and drug administration adverse event reporting system, database, signal processing

## Abstract

**Objective:** This study aims to explore the risk signals of osteonecrosis of the jaw induced by antiresorptive drugs and provide references for the clinical safety application.

**Method:** According to the FDA’s Adverse Event Reporting System (FAERS), from January 2004 to September 2021, we chose “Osteonecrosis of the jaw (10064658)” and “Exposed bone in jaw (10071014)” as preferred terms, “antiresorptive drugs” as the target drugs, and primary suspect drug as the drug role code in the dataset. We evaluated the association between drugs and adverse events by using reporting odds ratio (ROR) based on disproportionality analysis. We took the High-Level Terms (HLT) of MedDRA^®^ as the classification level of indications to calculate ROR to compare the signal difference of ONJ in different indications. In addition, patients with antiresorptive-induced osteonecrosis of the jaw and the time of onset of the condition following different antiresorptive medications were collected for the study.

**Results:** The FAERS contained 18,421 reports relating to jaw osteonecrosis from January 2004 to September 2021. A total of eight antiresorptive agents were included in the analysis. From high to low, the ROR of ONJ induced by antiresorptive agents (regardless of indication) is pamidronate (ROR = 494.8), zoledronic acid (ROR = 431.9), denosumab (ROR = 194.8), alendronate (ROR = 151.2), risedronate (ROR = 140.2), etidronic acid (ROR = 64.5), ibandronate (ROR = 40.8), and romosozumab (ROR = 6.4). HLT ROR values for “metabolic bone disorders” were the lowest for each drug, while HLT ROR values were high for “tumor-related indications,” including breast and nipple neoplasms malignant, plasma cell myelomas, and prostatic neoplasms malignant. The onset time for osteonecrosis of the jaw as median (Q1, Q3), osteoporosis-related indications, and the onset time for ONJ were 730 (368, 1268), 489.5 (236.3, 909.8), 722.5 (314, 1055), 761 (368, 1720), and 153 (50, 346) for zoledronic acid, denosumab, ibandronate, risedronate, and romosozumab, respectively. Cancer-related indications: the onset time for ONJ were 680.5 (255.3, 1283), 488 (245, 851), and 696.5 (347, 1087) for zoledronic acid, denosumab, and pamidronate, respectively.

**Conclusion:** When antiresorptive drugs are used for metastasis, they have the largest risk signal, followed by malignancy, and the smallest is osteoporosis. The onset time of ONJ may not be related to the indications. The onset time of ONJ for BPs was about 2 years, denosumab about 1.3 years, and romosozumab less than 1 year, which may be related to sequential treatment. When used according to the instructions, the risk of ONJ caused by denosumab was higher than that of zoledronic acid, regardless of the indication. Based on these findings, researchers will continue to monitor and identify risk factors.

## Introduction

Marx established an association between bisphosphonates (BPs) used for malignant bone diseases and avascular bone necrosis in the jaws in 2003 (Marx, 2003). Since then, several studies have been published on this disease, known as “bisphosphonate-related osteonecrosis of the jaws” (BRONJ). Research and medical communities have shown considerable interest in BRONJ, but the disease’s pathogenesis remains unclear. Various hypotheses of pathogenesis underlying BRONJ have been proposed. The most popular hypothesis is bone necrosis resulting from bisphosphonate-induced remodeling suppression (Allen and Burr, 2009).

Interestingly, human monoclonal antibodies, including denosumab and romosozumab, have different mechanisms of action from bisphosphonates. However, denosumab and romosozumab may also have an antiresorptive effect, raising concerns about ONJ development in patients. Denosumab, a monoclonal antibody fully human, has a high affinity and specificity for receptor activators of nuclear factor-B (RANK) ligand (RANKL), which is known to induce ONJ. Denosumab was found to cause ONJ in 1.8% of patients receiving it over 3 years in pivotal phase III trials in patients with solid tumor bone metastases (Pittman et al., 2017). In addition, it has been shown that denosumab suppresses bone turnover as well or better than BPs (Uyanne et al., 2014). Based on randomized clinical trials (RCTs), the same meta-analysis determined that denosumab therapy led to a higher risk of ONJ than BP therapy (Boquete-Castro et al., 2016). In addition to the above drugs, romosozumab, a humanized monoclonal antibody that targets sclerostin, is approved for osteoporosis treatment (European Medicines Agency, 2019). During the phase III (FRAME) study of osteoporosis patients, two cases of ONJ were reported as positive (Cosman et al., 2016; Paik and Scott, 2020). Recent experiments on mice showed that romosozumab could not cause ONJ-like lesions (Hadaya et al., 2019).

Most reports of ONJ occur in patients receiving high-dose intravenous (IV) BPs because of malignant diseases such as cancer, prostate, or multiple myeloma (Hansen et al., 2013). When patients take BRONJ for osteoporosis, the incidence is much lower than when they take it for malignant diseases (Dimopoulos et al., 2006). Approved indications of antiresorptive drugs are different. Table 1 presents a list of antiresorptive drugs approved in the United States and their indications. Most commonly, BPs are administered orally or intravenously. Alendronate, ibandronate, and risedronate are the most common BPs administered orally. Patients with osteopenia, osteoporosis, and Paget’s disease can benefit from oral administration of BPs. Intravenous administration of BPs is commonly used for treating bone metastases caused by solid tumors, multiple myeloma, and malignancy-related hypercalcemia. Approximately 0.04% of patients receiving oral BPs contract ONJ, compared with 5%–20% for those receiving IV BPs. (Edwards et al., 2009). Over 90% of all prescriptions for bisphosphonates are for osteoporosis. Globally, as life expectancies increase, more people live past 65 years old (Crews and Zavotka, 2006). Consequently, the number of older people and patients with osteoporosis and cancer is likely to increase, increasing the use of antiresorptive agents.

**TABLE 1:** Antiresorptive agents approved in the United States, in order of year first marketed.

Indications drugs	Osteoporosis in postmenopausal women	Osteoporosis in men	Glucocorticoid-induced osteoporosis	Paget” s disease of bone	Multiple myeloma and bone metastasis from solid tumors	Hypercalcemia of malignancy	Giant cell tumor of bone	Brand name	Year of first marketed
Etidronate	N	N	N	Y	N	N	N	Didronel	09/01/1977
Pamidronate	N	N	N	Y	Y	Y	N	Aredia	10/31/1991
Alendronate	Y	Y	Y	Y	N	N	N	Fosamax	09/29/1995
Risedronate	Y	Y	Y	Y	N	N	N	Actonel	03/27/1998
Zoledronic Acid	Y	Y	Y	Y	Y	Y	N	Zometa	8/20/2001
Ibandronate	Y	N	N	N	N	N	N	Boniva	05/16/2003
Denosumab	Y	Y	Y	Y	Y	Y	Y	Xgeva	06/01/2010
Romosozumab	Y	N	N	N	N	N	N	Evenity	04/09/2019

Do the drugs cause a difference in ONJ risk due to different indications? How long can ONJ occur after taking the medicines? The risk of the number of ONJ patients worldwide is also increasing. All of the above issues need further study.

Because ONJ is a rare ADR, RCTs are difficult to conduct. However, it is becoming increasingly common to use data-mining techniques to examine and analyze large amounts of accumulated data from medical databases to identify how drugs relate to adverse events (Harpaz et al., 2013). Submitted by the public, the FDA Adverse Event Reporting System (FAERS) is a voluntary, spontaneous reporting system (SRS) that provides FDA post-marketing safety information for approved drugs and biologics (Sakaeda et al., 2013). In this pharmacovigilance study, multiple details were collected from a large real-life dataset further to characterize the profile of antiresorptive drug treatments with ONJ.

## Material and methods

### Data source

Data from the FAERS database from January 2004 to September 2021 were used for a retrospective pharmacovigilance study. The FDA’s Adverse Event Reporting System (FAERS) includes reports of adverse events, medication errors, and complaints about product quality resulting in adverse events and submitted to the FDA by healthcare professionals, manufacturers, consumers, and patients. In addition, each report contains information about demographics, drugs, and reactions in the database. Each report has a primary suspected drug and may contain information about other drugs taken by the patient (Food and Drug Administration, 2021)*.*

### Data mining

**FIGURE 1:**
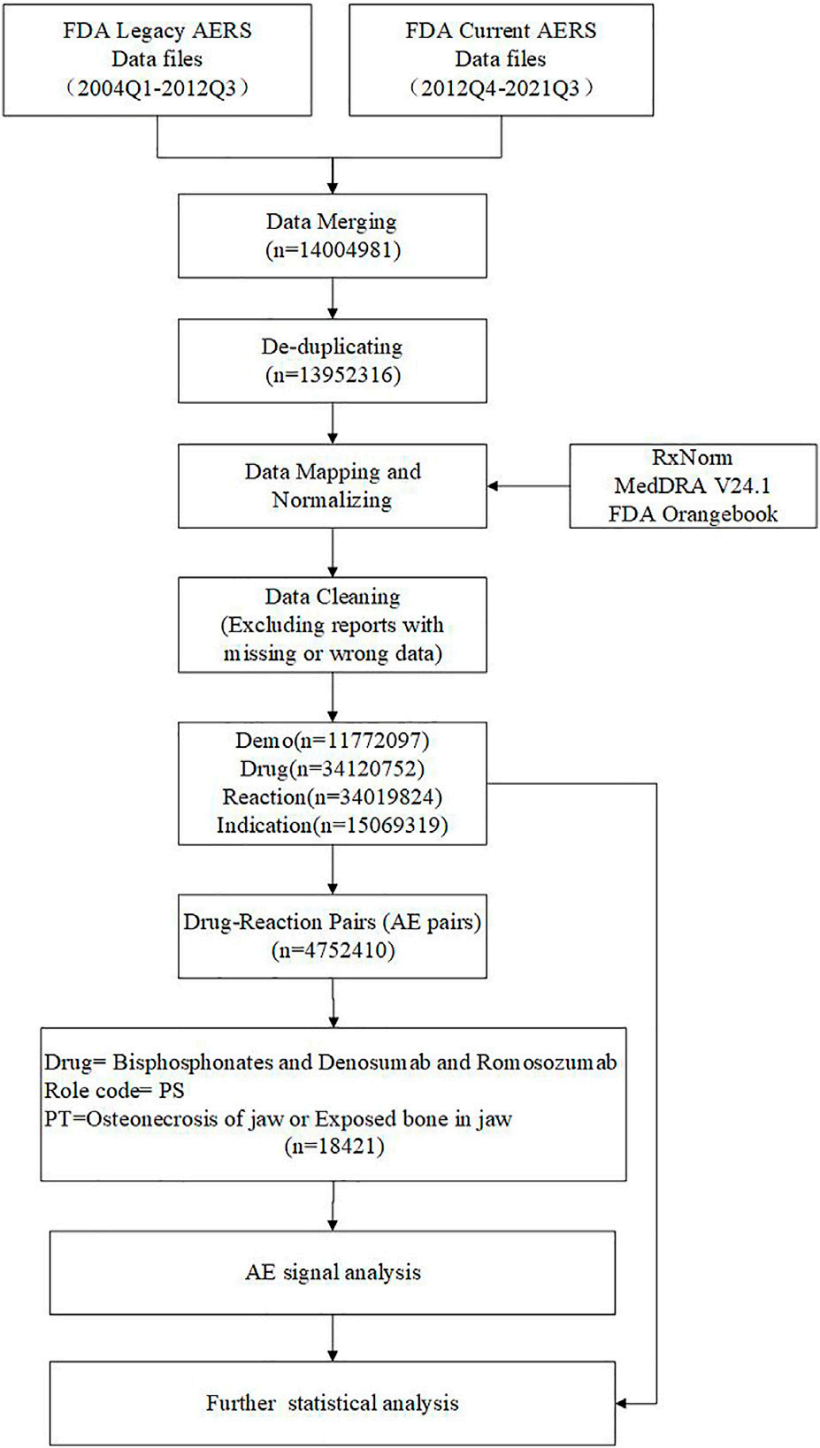
Flow diagram of data extraction and cleaning.

FAERS data requires substantial curated cleaning and normalizing before they can be used appropriately. Otherwise, data can have a massive impact on analysis results. We used Python (version 3.8) and Postgresql (version 14) to handle the cleaning and normalizing process, which included merging data, de-duplicating records, applying standardized vocabularies with drug names mapped to RxNorm concepts, indications, and outcomes mapped to the systematized nomenclature of human and veterinary medicine clinical terms (SNOMED-CT) concepts, normalizing reaction, and indication to Medical Dictionary for Regulatory Activities (MedDRA^®^) (version 24.0) concepts and used R software (version 4.1.0) to statistical compute drug-reaction signals. Based on disproportionality analysis, we used reporting odds ratio (ROR) to investigate the potential signals between the drug and the specific adverse event of interest. The calculation and criteria of the algorithm are reported (Van Puijenbroek et al., 2002; Hauben et al., 2005) as follows: ROR = $\frac{ad}{bc}$ , $95\%\;CI=e^{\ln\left(ROR\right)\pm 1.96\sqrt{\frac{1}{a}+\frac{1}{b}+\frac{1}{c}+\frac{1}{d}}}$ (Criteria: a≥3, the lower 95% CI > 1). In the algorithm, a represents how many reports contain a suspect drug and a suspected adverse reaction to that drug; b is the number of reports that contain suspected adverse drug reactions with other medications (other than the drug of interest); in addition to the event of interest, c is the number of reports relating to the suspect drug with other adverse drug reactions (other than the event of interest); d is the number of reports containing other medications and other adverse drug reactions. In addition to calculating the ROR of ONJ induced by the drugs (regardless of indications), we took the High-Level Terms (HLT) of MedDRA as the classification level of indications to calculate ROR to compare the signal difference of ONJ in different indications. Through the above steps, our total dataset was formed. The Prisma diagram is shown in Figure 1.

We chose “Osteonecrosis of the jaw (10064658)” and “Exposed bone in jaw (10071014)” as Preferred Terms (PT), with the drug role code as “PS” (Primary Suspect Drug) in the total dataset. Cases fulfilling ROR criteria were recognized as a signal, analyzed, and as a case group. If the ROR in the control group is meaningful, we believe there is a statistical significance between the drug and ADR, i.e., drugs risk causing ONJ. If the drug is only relevant in the case group, we believe it may not always cause the ADR. A higher ROR suggested a stronger reporting relationship between the ONJ and the antiresorptive agents.

### Statistical analysis

Categorical variables are reported as frequency and percentage to summarize the clinical characteristics of ONJ from the FAERS database. The age of reporting cases and onset time of ONJ are shown as median values with 25% and 75% quartiles, expressed as median (Q1, Q3). The Mann-Whitney test was used to compare the onset time difference of the same drug for different indications. The statistical significance level was set at *p* ＜ 0.05.

## Results

### Descriptive analysis

**FIGURE 2:**
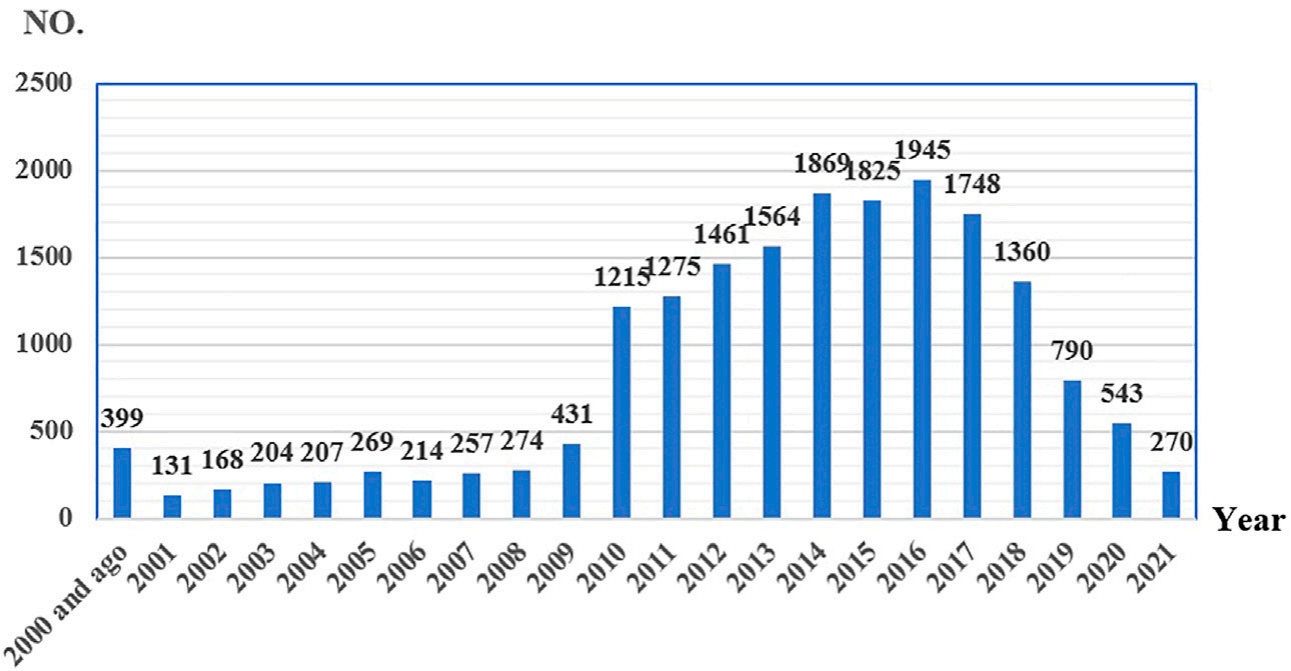
Reporting years of antiresorptive-related to osteonecrosis of the jaw collected from FAERS (number of reports).

From 2004 to September 2021, there were 13,987,127 AE reports in the FAERS database. About 5,444,088 adverse drug event combinations and signal values were formed after data cleaning and calculation. Eight antiresorptive agents were included in the analysis. We screened 18,421 reports with suspected antiresorptive-related osteonecrosis of the jaw and summarized the clinical features of these patients in Table 2. Zoledronic acid and denosumab were reported more than other agents. There was a tendency for affected patients to be older than 65. In this study, indications of antiresorptive drugs were combined for more than 1% of FAERS. We merged osteopetrosis, postmenopause, osteoporosis prophylaxis, and osteopenia into “Osteoporosis-related” and merged metastases to bone, prostate cancer, breast cancer, and neoplasm malignant into “Cancer-related.” Different drugs have different indications, and the signal of ONJ may differ. Alendronate (98.17%), risedronate (95.36%), etidronate (100.00%), romosozumab (100.00%), and ibandronate (74.52%) were mainly used in patients with osteoporosis, while zoledronic acid (90.02%), pamidronate (88.24%), and denosumab (62.87%) were mainly used in patients with tumors. The clinical outcomes caused by different drugs were similar. Health-professional were the leading reporters. The main reporting regions were different for different drugs. Cases of zoledronic acid were mainly reported from Europe (51.23%). Alendronate, denosumab, and risedronate were mainly reported from Europe and North America. Cases of romosozumab were mainly reported from South America. The reported cases of antiresorptive-related osteonecrosis of the jaw gradually increased from 2004 to 2016 but showed a downward trend after 2017. See Figure 2.

**TABLE 2:** Detailed information on antiresorptive-related osteonecrosis of jaw reports.

		Zoledronic acid N (%)	Alendronate, N (%)	Denosumab, N (%)	Risedronate, N (%)	Ibandronate, N (%)	Pamidronate, N (%)	Romosozumab, N (%)	Etidronate, N (%)
Gender	F	4149 (54.49)	2025 (80.42)	4095 (57.07)	189 (86.30)	420 (87.32)	258 (65.98)	16 (88.89)	2 (66.67)
	M	2975 (39.07)	249 (9.89)	2079 (28.98)	19 (8.68)	30 (6.24)	99 (25.32)	1 (5.56)	1 (33.33)
	Unknown	490 (6.44)	244 (9.69)	1001 (13.95)	11 (5.02)	31 (6.44)	34 (8.70)	1 (5.56)	
Age	N	5994	1855	4648	155	357	150	901	2
	Median (Q1, Q3)	66 (58, 74)	69 (60, 77)	71 (64, 78)	73 (66, 81)	73 (63, 80)	65 (59, 75)	65 (55,75)	60 (60, 60)
Continent	North America	1468 (19.28)	1508 (59.89)	2193 (30.56)	43 (17.55)	0	14 (3.58)	0	0
	Oceania	59 (0.77)	3 (0.12)	214 (2.98)	2 (0.82)	4 (0.83)	8 (2.05)	0	0
	Africa	22 (0.29)	0	2 (0.03)	1 (0.41)	250 (51.98)	100 (25.58)	1 (5.56)	
	South America	178 (2.34)	6 (0.24)	61 (0.85)	86 (35.10)	62 (12.89)	26 (6.65)	12 (66.67)	1 (33.33)
	Europe	3901 (51.23)	832 (33.04)	3186 (44.40)	64 (26.12)	2 (0.42)	2 (0.51)	0	0
	Asia	1764 (23.17)	158 (6.27)	1516 (21.13)	7 (2.86)	0	0	0	0
	Unknown	222 (2.92)	11 (0.44)	3 (0.04)	42 (17.14)	163 (33.89)	241 (61.64)	5 (27.78)	2 (66.67)
Reporter	Physician	1477 (19.40)	814 (32.33)	4254 (59.29)	17 (7.20)	191 (39.71)	35 (8.95)	15 (83.33)	2 (66.67)
	Other health-professional	4473 (58.74)	954 (37.89)	1490 (20.77)	90 (38.09)	95 (19.75)	135 (34.53)	0	1 (33.33)
	Consumer	939 (12.33)	422 (16.76)	1025 (14.29)	30 (12.71)	52 (10.81)	0	1 (5.56)	0
	Pharmacist	134 (1.76)	36 (1.43)	209 (2.91)	26 (11.02)	10 (2.08)	4 (1.02)	0	0
	Lawyer	63 (0.83)	108 (4.29)	6 (0.08)	9 (3.81)	61 (12.68)	203 (51.92)	0	0
	Unknown	528 (6.93)	184 (7.31)	191 (2.66)	64 (27.12)	72 (14.97)	14 (3.58)	2 (11.11)	0
Indications	Tumor and Tumor-related	4963 (90.02)	35 (1.83)	2877 (62.87)	7 (4.64)	92 (25.48)	105 (88.24)	0 (0.00)	0 (0.00)
	Osteoporosis-related	550 (9.98)	1877 (98.17)	1699 (37.13)	144 (95.36)	269 (74.52)	14 (11.76)	16 (100.00)	1 (100.00)
Outcomes	Death	604 (6.58)	87 (2.05)	213 (2.54)	5 (1.69)	8 (1.48)	14 (2.30)	0 (0.00)	0 (0.00)
	Disability	665 (7.25)	824 (19.44)	355 (4.24)	20 (6.76)	40 (7.38)	205 (33.61)	1 (4.55)	1 (4.55)
	Hospitalization - Initial or Prolonged	1581 (17.23)	1123 (26.49)	989 (11.80)	84 (28.38)	110 (20.30)	26 (4.26)	4 (18.18)	4 (18.18)
	Life-Threatening	53 (0.58)	24 (0.57)	34 (0.41)	9 (3.04)	7 (1.29)	2 (0.33)	0 (0.00)	0 (0.00)
	Other Serious (Important Medical Event)	6272 (68.36)	2181 (51.45)	6789 (81.01)	178 (60.14)	377 (69.56)	363 (59.51)	17 (77.27)	17 (77.27)

N, number of reports containing the suspect drug and the suspect adverse drug reaction. Associations of different antiresorptive agents with osteonecrosis of the jaw.

### Disproportionality analysis

**FIGURE 3:**
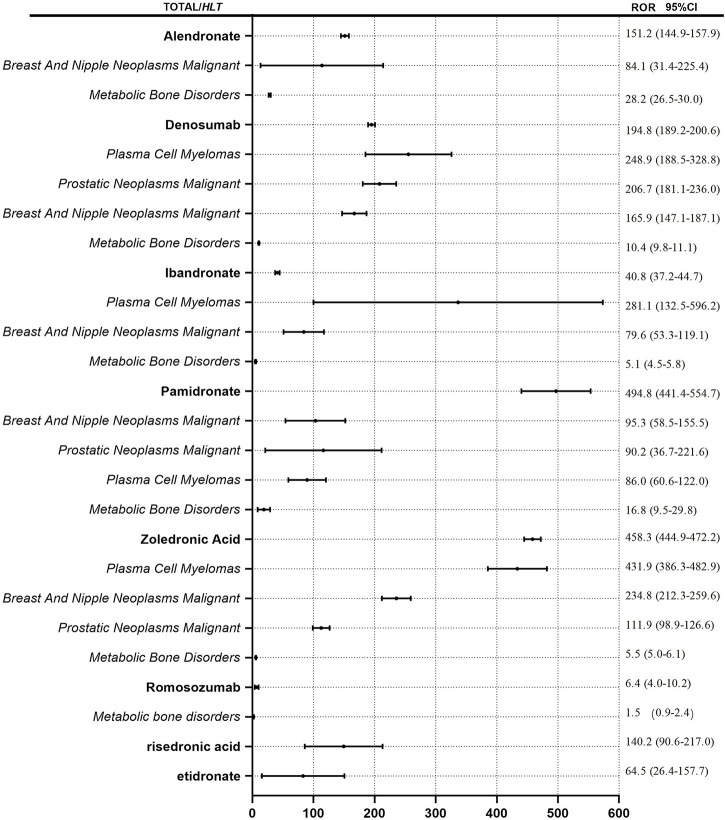
ROR for ONJ with antiresorptive drugs. Notes: ROR values for drug names is calculated regardless of indications. ROR values for the italics is calculated by different indications.

We detected signals of osteonecrosis of the jaw for all eight antiresorptive agents based on the criteria for ROR and showed the results in Figure 3. ROR of ONJ induced by the antiresorptive agents (regardless of indications) from high to low in turn is pamidronate (ROR = 494.8), zoledronic acid (ROR = 431.9), denosumab (ROR = 194.8), alendronate (ROR = 151.2), risedronate (ROR = 140.2), etidronic acid (ROR = 64.5), ibandronate (ROR = 40.8), and romosozumab (ROR = 6.4). We took the HLT of MedDRA^®^ as the classification level of indications to calculate ROR. HLT analyses of risedronate and etidronate were not conducted due to the low number of reports. “Metabolic bone disorders, breast and nipple neoplasms malignant, plasma cell myelomas, and prostatic neoplasms malignant” were included in HLT for denosumab, pamidronate, and zoledronic acid. Metabolic bone disorders, breast and nipple neoplasms malignant, and plasma cell myelomas were included in HLT for alendronate and ibandronate. Metabolic bone disorders were included in HLT for romosozumab. It is worth mentioning that the ROR of plasma cell myeloma as HLT for alendronate was 1375.4, and lower and upper limits of 95% CI for ROR were 277.4 and 6820.3. This outcome is not found in Figure 3. The data was too large to be shown well. As demonstrated in Figure 3, the ROR value for each drug is minimum when HLT is “metabolic bone disorders” for each drug. ROR was less than 30 for each drug. However, when HLT is tumor-related indications, “breast and nipple neoplasms malignant, plasma cell myelomas and prostatic neoplasms malignant,” ROR value increases. The maximum ROR value is 1375.4.

**FIGURE 4:**
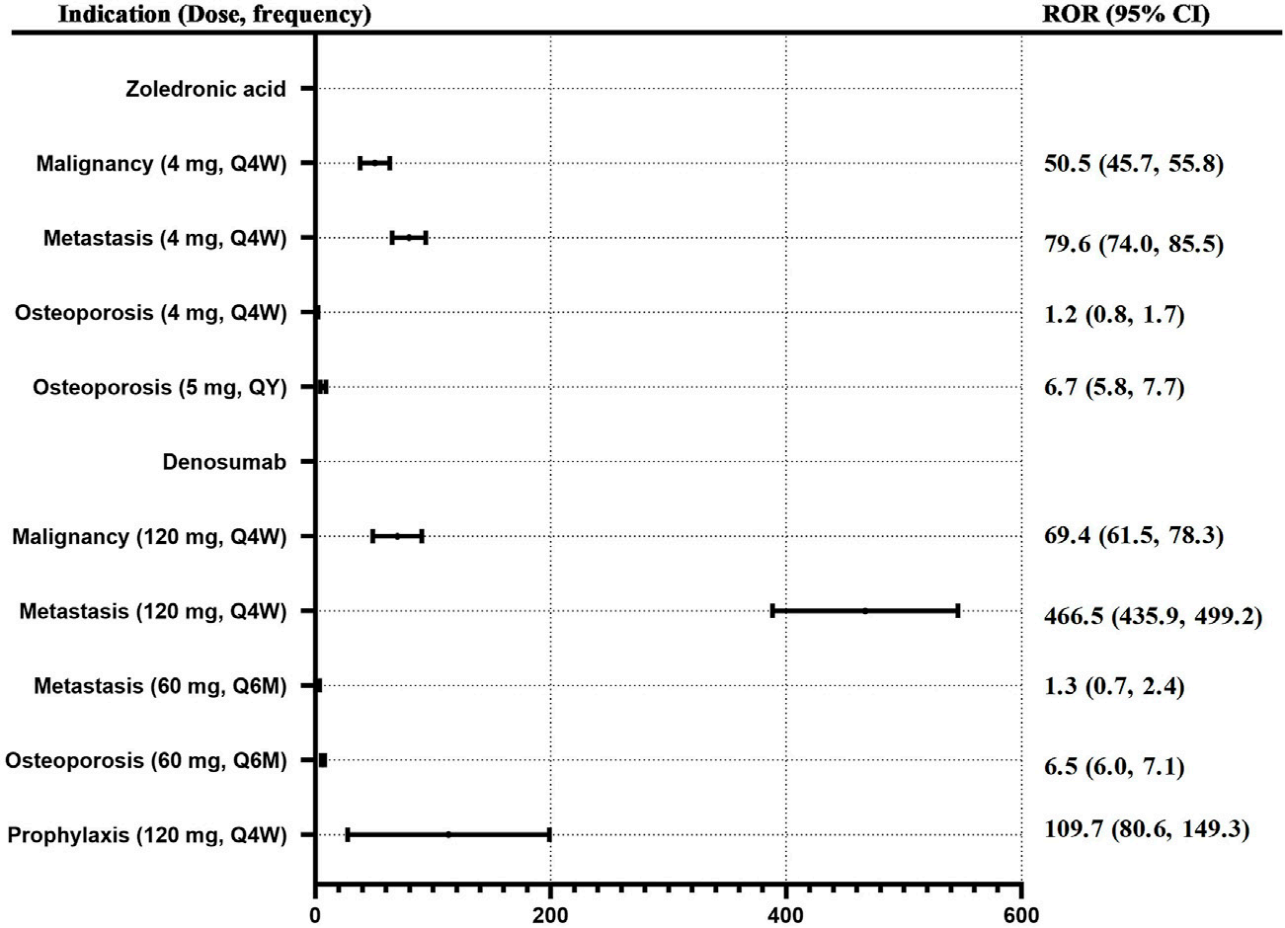
ROR for ONJ of denosumab and zoledronic acid with different doses and frequency for different indications. Note: Q4W, every 4 weeks; Q6M, every 6 months, QY, every 1 year.

Based on the original data, the data of dose-frequency-indication was matched and extracted. All cases with missing doses, frequencies, or indications were excluded. Finally, 5,368 cases were included in the analysis. Except for prophylaxis solely, similar indications were combined. Osteopetrosis, osteoporosis postmenopause, osteoporosis prophylaxis, and osteopenia were merged into “Osteoporosis,”; merged prostate cancer, breast cancer, and neoplasm malignant into “Malignancy”; merged metastases to bone, metastases to breast, metastases to prostate, breast cancer metastatic, prostate cancer metastatic, metastasis, and metastatic neoplasm into “Metastasis”; and different doses were calculated separately. ROR values for ONJ of denosumab and zoledronic acid with different doses and frequencies for different indications were calculated. See Figure 4. When zoledronic acid (4 mg, Q4w) was used in metastasis, malignancy, and osteoporosis, the ROR (95% CI) was 79.6 (74.0, 85.5), 50.5 (45.7, 55.8), and 1.2 (0.8, 1.7) respectively. When denosumab (120 mg, Q4w) was used in metastasis, malignancy, and prophylaxis, the ROR (95% CI) was 466.5 (435.9, 499.2), 69.4 (61.5, 78.3), and 109.7 (80.6, 149.3) respectively; When denosumab (60 mg, Q6m) was used in metastasis and osteoporosis, the ROR (95% CI) was 1.3 (0.7, 2.4) and 6.5 (6.0, 7.1) respectively.

### Time to onset of antiresorptive-related osteonecrosis of the jaw

**FIGURE 5:**
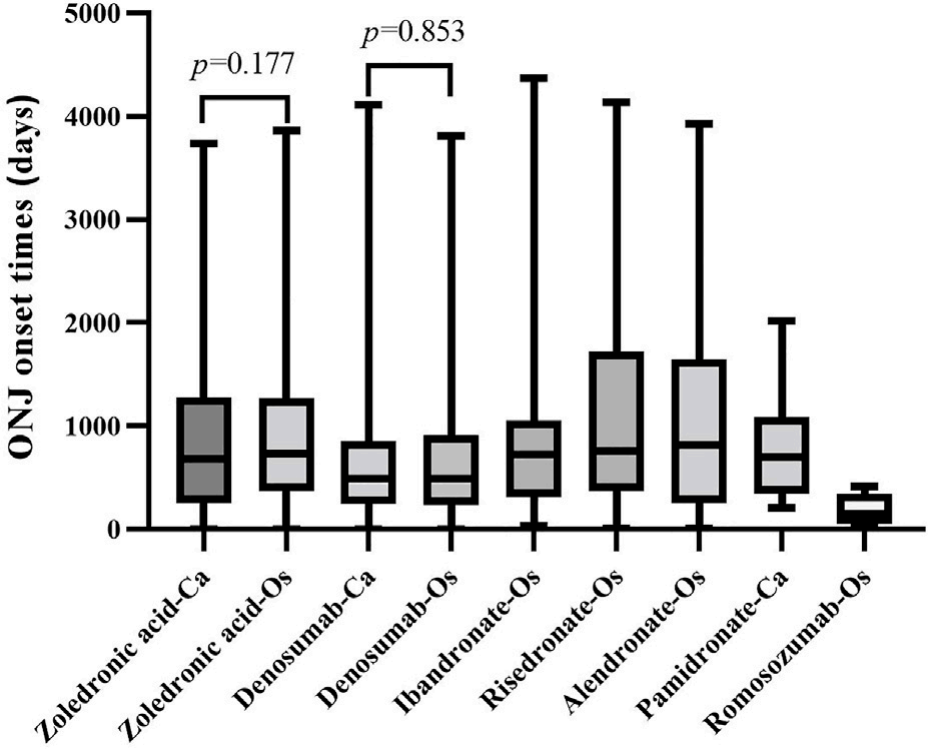
The onset time of ONJ induced by the antiresorptive drug with different indications. Abbreviation: ONJ, osteonecrosis of the jaw; Os, osteonecrosis-related indications; Ca, cancer-related indications

According to the combined indications, the onset time for ONJ induced by antiresorptive drugs in “osteoporosis-related” and “cancer-related” indications were calculated separately and shown as median (Q1, Q3) (Figure 5). Osteoporosis-related indications, the onset time for ONJ were 730 (368, 1268), 489.5 (236.3, 909.8), 722.5 (314, 1055), 761 (368, 1720), and 153 (50, 346) for zoledronic acid, denosumab, ibandronate, risedronate, romosozumab respectively; cancer-related” indications, the onset time for ONJ were 680.5 (255.3, 1283), 488 (245, 851), and 696.5 (347, 1087) for zoledronic acid, denosumab, pamidronate respectively.

The Mann-Whitney test was used to compare the onset time difference of zoledronic acid, denosumab, and ibandronate for different indications. When zoledronic acid and denosumab were used in osteoporosis and cancer-related indications, there was no significant difference in the onset time (*p* > 0.05).

## Discussion

Using the FAERS pharmaceutical database, this study describes the difference in associations, timing, and prognosis of ONJ following antiresorptive drug use, and it is one of the largest collections of cases of this type in history. The study showed that all eight studied antiresorptive agents were associated with an adverse event of ONJ. However, there were distinctions across antiresorptive agents.

### Comparison of risk signals of antiresorptive drugs induced osteonecrosis of the jaw

Bone metastasis from solid tumors, breast cancer, multiple myeloma, and bone metastases from malignant may be treated or prevented with antiresorptive agents (Marx, 2003; Allen and Burr, 2009). In the pharmacovigilance investigation, alendronic acid, ibandronate, risedronate, romosozumab, and etidronate were mainly used for osteoporosis, with ROR values of 151.2, 40.8, 140.2, 6.5, and 64.5, respectively. Pamidronate and zoledronic acid were mainly used for tumor-related diseases, and their ROR values were 494.8 and 458.3, respectively. Denosumab was used in osteoporosis and tumor-related diseases, with a ROR value of 194.8. It can be seen that the indications of antiresorptive drugs induced ONJ are consistent with the approved indications. The problem has since attracted increasing attention, particularly among those who receive intravenous bisphosphonates for cancer-related conditions and those using oral osteoporosis preparations. Interestingly, the risk signals of ONJ are similar for drugs under the same indication. To further study the risk of ROR in different indications, we took the High-Level Terms (HLT) of MedDRA^®^ as the classification level of indications to calculate ROR. Consistent with the above conclusions, ROR values of HLT as “metabolic bone disorders” were the lowest for each drug, while HLT is tumor-related indications “breast and nipple neoplasms malignant, plasma cell myelomas and prostatic neoplasms malignant,” ROR value increases. This study supports other studies’ findings (Khosla et al., 2007). Patients with osteoporosis or Paget’s disease of the bones are less likely to experience ONJ. This is because patients with advanced malignancies receive higher doses and more frequent administration of antiresorptive agents than those with benign bone diseases. For example, cancer patients are given pamidronate or zoledronate intravenously every 3–4 weeks, at a dose that is 4–10 times higher than what is used to treat osteoporosis (Watts and Marciani, 2008). In order to further evaluate the relationship between dose-frequency-indication and ONJ occurrence, the dose-frequency-indication was matched and extracted based on the original data. It is only by using zoledronic acid or denosumab that enough data can be extracted for calculation. ROR values for ONJ of denosumab and zoledronic acid with dose-frequency-indication were calculated. When the same dose of zoledronic acid (4 mg, Q4w) was used for different indications, ROR values for ONJ induced by zoledronic acid for different indications were metastasis ＞ malignancy ＞ osteoporosis in order. When denosumab (120 mg, Q4w) was used in metastasis and malignancy, ROR values were also metastasis ＞ malignancy. There is a strong association between ONJ and underlying bone disease. However, when denosumab (6 mg, Q6m) was used in metastasis and osteoporosis, ROR values were metastasis ＜ osteoporosis. Considering data of denosumab (6 mg, Q6m) used in metastatic was small. When the indications are consistent, the ROR value of ONJ is denosumab > zoledronic acid. Ehrenstein et al. (2021) conducted a clinical trial with cancer patients treated with denosumab- or zoledronic acid in Denmark, Norway, and Sweden. In this study, among denosumab-treated patients, ONJ developed in 5.7%; zoledronic acid-treated patients in 1.4%. In our study, when the indication is malignancy, the ROR values of ONJ for denosumab versus zoledronic acid were similar. However, when the indication is metastasis, the ROR value of ONJ for denosumab (120 mg, Q4w) versus zoledronic acid (4 mg, Q4w) was 466.5 versus 79.6, similar to the clinical study. In our study, when osteoporosis is indicated, the ROR value of ONJ for denosumab (60 mg, Q6m) versus zoledronic acid (5 mg, Qy) was 6.5 versus 6.7. This result was inconsistent with the study to evaluate the incidence of ONJ among osteoporosis patients treated with denosumab *versus* bisphosphonates (BPs). Everts-Gra et al. (Everts-Graber et al., 2022) concluded that the rate ratio of 6.3 (95% CI: 2.1–22.8) in patients receiving denosumab *versus* patients receiving BP-associated ONJ is significant. Denosumab was associated with 28.3 ONJ per 10′000 observed patient-years, while BP-associated ONJ was associated with 4.5 ONJ per 10′000 observed patient years. In our study, zoledronic acid was mainly (90.02%) used for cancer-related indications. The data on zoledronic acid used for osteoporosis-related indications was small. Especially the data matched dose frequency. Above all, we agree that there is a strong association between ONJ and the underlying bone disease and antiresorptive regimens used in treating it (Anastasilakis et al., 2022).

The risk of ONJ between alendronate, ibandronate, risedronate, and romosozumab in the treatment of osteoporosis differs with gender. The proportion of ONJ in women is more than 80%. Considering osteoporosis is less prevalent in men than women, for example, among Taiwanese women and men over 50, osteoporosis prevalence averaged 11.4% and 1.6%, respectively (Wang et al., 2009). Therefore, women have a greater chance to prescribe such drugs. On the other hand, when drugs were used in cancer patients, the proportion of women decreased. For example, the proportion of women with zoledronic acid was 54.49%, and denosumab was 57.07%. One interesting finding was that the rate of antiresorptive-related osteonecrosis of the jaw in males was comparable to that in females with the indications for cancer-related treatment. So, we could not conclude that the female sex is a risk factor for ONJ in cancer patients or osteoporosis patients.

### Time to onset of antiresorptive-related osteonecrosis of the jaw

The pharmacovigilance investigation calculated the onset time for ONJ induced by antiresorptive drugs in “osteoporosis-related” and “cancer-related” indications separately. Osteoporosis-related indications, the onset time for ONJ was about 2 years for zoledronic acid, ibandronate, and risedronate, about 1.3 years for denosumab, and 0.5 years for romosozumab, similar to cancer-related. In our study, the onset time of ONJ caused by antiresorptive drugs may have nothing to do with the disease itself. However, we have to admit that there may be inaccuracies in calculating the onset time of ONJ in FAERS data. The duration of the phenomenon also varies, with some studies putting it at 10–18 months during zoledronate treatment and 1.5–2.8 years in the case of pamidronate. It is also considered related to the drug dosage form. Alendronate, etidronate, and risedronate are oral preparations. Oral drugs may take longer to produce ONJ. Consistent with our record, a study (Nakamura et al., 2015) showed that the mean time to ONJ after zoledronate treatment was calculated at 1.8 years. After pamidronate, the mean time was 2.8 years. In our study, the onset time for ONJ induced by denosumab, especially romosozumab, was shorter than BPs, considering it may be related to the fact that these two drugs may be used as a sequential treatment for BPs. The database may only retain the last drug used as the primary suspect drug, and the use of other drugs may not be fully collected.

Although two cases of ONJ were adjudicated in the phase III FRAME study of women with osteoporosis, the development of ONJ with romosozumab in a recent experimental study in mice could not be replicated. Our study found positive signals of romosozumab in FAERS, but encouragingly, this drug causes the slightest risk signal for ONJ. It should be noted that the onset time of ONJ induced by romosozumab is 169.5 days, which is shorter than that of other antiresorptive drugs. Humanized monoclonal antibody romosozumab inhibits sclerostin, which is involved in bone resorption and promotes bone formation. Several countries have approved subcutaneous romosozumab, including the United States for osteoporosis in postmenopausal women at high risk of fractures and the EU for treating severe osteoporosis (Paik and Scott, 2020). Therefore, when the drug is used in the clinical setting, it is necessary to pay more attention to the possible earlier ONJ risk (Migliorati et al., 2019).

Marx established an association between bisphosphonates (BPs) used for malignant bone diseases and avascular bone necrosis in the jaws in 2003 (Marx, 2003). Since then, numerous studies have been published concerning the disease. For the reporting of ONJ, the MedDRA^®^ preferred term “osteonecrosis of jaw” was established in March 2010 (www.meddra.org). Since 2010, the number of ONJ reports has increased significantly. The risk factors of ONJ were also gradually revealed. Risk factors associated with the development of ONJ include poor oral hygiene, bone invasive dental procedures, such as tooth extraction or dentoalveolar surgery, comorbidity conditions (most notably cancer), lifestyle, and behaviors (tobacco, alcohol) (Almazrooa and Woo, 2009; Kuroshima et al., 2019). However, the number of ONJ reports began to decrease in 2017. It is speculated that the risk and the prevention of ONJ have gradually attracted clinical attention. We believe that the risk of antiresorptive-related ONJ can be reduced through effective prevention.

This study has several advantages due to the real-world research and data mining techniques used, but it also has some disadvantages (Zhao et al., 2021), such as underreporting, incomplete reporting, false reporting, inaccuracy, and arbitrariness. Second, the absence of the total number of patients receiving treatment makes it impossible to calculate relevant statistics, such as the incidence rate for each suspicious drug. Third, it is challenging to propose specific important risk factors between drugs and ONJ due to a lack of information. Fourth, FAERS-based pharmacovigilance studies have limitations due to the SRS principle described above. However, in this study, we have identified signals between antiresorptive drugs and ONJ, which may provide clues for further and better-organized studies of antiresorptive-related ONJ. Notably, this study provided guidance and inspiration for developing antiresorptive drugs for ONJ and other disorders.

## Data Availability

The original contributions presented in the study are included in the article/supplementary materials, further inquiries can be directed to the corresponding authors.
